# Compliance of hand hygiene of healthcare workers in emergency care unit in a private tertiary hospital in São Paulo.

**DOI:** 10.1186/2047-2994-4-S1-P158

**Published:** 2015-06-16

**Authors:** EA Tito, J Kawagoe, P Gonçalves

**Affiliations:** 1 Hospital Israelita Albert Einstein, São Paulo, Brazil

## Background

The objective of this study was to assess the compliance of hand hygiene (HH) of healthcare workers (HCWs) in emergency care unit in a private tertiary hospital in São Paulo.

## Methods

An observational study was conducted on the compliance of HH for the five World Health Organization (WHO) indications. HCWs were observed during routine patient care in day shift. The authors also measured the technique of HH through hand washing or hand hygiene with alcohol-based disinfectant. An observational study was performed before and after intervention. This intervention did training with the multidisciplinary team and communication (posters, email, phone and vídeo). The video was done with the healthcare workers in emergency care unit.

## Results

A total of 530 HH opportunities were identified during the observation period. Overal compliance before intervention (BI) was 56% and after intervention (AI) 72,4% (P<0.05). Compliance before and after intervention: nurses (52,1% and 68.3%)[p < 0.05] and doctors (59% and 75%) [p< 0.05].

## Conclusion

Adherence to hand hygiene practice and use of alcohol-based disinfectant of the doctor was high compared with the literature. The main point was the participation of physicians in the intervention.

## Disclosure of interest

None declared.

